# Circulating microbiome analysis in patients with perioperative anaphylaxis

**DOI:** 10.3389/fimmu.2023.1241851

**Published:** 2024-01-11

**Authors:** Luc de Chaisemartin, Dragos Ciocan, Aurélie Gouel-Chéron, Vanessa Granger, Dan Longrois, Philippe Montravers, Anne-Marie Cassard, Sylvie Chollet-Martin

**Affiliations:** ^1^ AP-HP, Immunology Department, Bichat Hospital, Paris, France; ^2^ Université Paris-Saclay, Inserm, Inflammation, Microbiome, Immunosurveillance, Orsay, France; ^3^ AP-HP, Hepatogastroenterology and Nutrition, Hôpital Antoine-Béclère, Clamart, France; ^4^ Département d’Anesthésie-Réanimation, CHU Bichat-Claude Bernard, DMU PARABOL, AP-HP.Nord, AP-HP, Paris, France; ^5^ Institut Pasteur, Antibodies in Therapy and Pathology, Inserm UMR 1222, Paris, France; ^6^ Division of Clinical Research, National Institute of Allergy and Infectious Diseases, National Institutes of Health, Bethesda, MD, United States; ^7^ Université de Paris, FHU PROMICE, Paris, France; ^8^ Anaesthesiology and Critical Care Medicine Department, DMU PARABOL, Bichat-Claude Bernard and Louis Mourier Hospitals, AP-HP, Paris, France; ^9^ INSERM UMR 1148, Atherothrombotic Disease in Heart and Brain, Paris, France; ^10^ Université Paris Cité, Inserm, PHERE, Paris, France; ^11^ Paris Center for Microbiome Medicine (PaCeMM) FHU, Paris, France

**Keywords:** IgE-mediated hypersensitivity, microbiome, neuromuscular blocking agents, perioperative anaphylaxis, tryptase

## Abstract

**Background:**

Perioperative anaphylaxis is a rare and acute systemic manifestation of drug-induced hypersensitivity reactions that occurs following anesthesia induction; the two main classes of drugs responsible for these reactions being neuromuscular blocking agents (NMBA) and antibiotics. The sensitization mechanisms to the drugs are not precisely known, and few risk factors have been described. A growing body of evidence underlines a link between occurrence of allergy and microbiota composition. However, no data exist on microbiota in perioperative anaphylaxis. The aim of this study was to compare circulating microbiota richness and composition between perioperative anaphylaxis patients and matched controls.

**Methods:**

Circulating 16s rDNA was quantified and sequenced in serum samples from 20 individuals with fully characterized IgE-mediated NMBA-related anaphylaxis and 20 controls matched on sex, age, NMBA received, type of surgery and infectious status. Microbiota composition was analyzed with a published bioinformatic pipeline and links with patients clinical and biological data investigated.

**Results:**

Analysis of microbiota diversity showed that anaphylaxis patients seem to have a richer circulating microbiota than controls, but no major differences of composition could be detected with global diversity indexes. Pairwise comparison showed a difference in relative abundance between patients and controls for *Saprospiraceae, Enterobacteriaceae, Veillonellaceae, Escherichia-Shigella, Pseudarcicella, Rhodoferax*, and *Lewinella*. Some taxa were associated with concentrations of mast cell tryptase and specific IgE.

**Conclusion:**

We did not find a global difference in terms of microbiota composition between anaphylaxis patient and controls. However, several taxa were associated with anaphylaxis patients and with their biological data. These findings must be further confirmed in different settings to broaden our understanding of drug anaphylaxis pathophysiology and identify predisposition markers.

## Introduction

Anaphylaxis is a rare and acute systemic manifestation of type I hypersensitivity that can be life-threatening. The most common triggers of anaphylaxis are food in children and drugs as well as insect venoms in adults. Classically, the anaphylaxis mechanism involves histamine degranulation by mast cell upon cross-binding of surface-bound specific IgE by the allergen. However, several additional mechanisms have been described by us and others involving other components of the immune system like IgGs, neutrophils and complement (1, 2). One of the main anaphylaxis settings in adults is perioperative anaphylaxis. These acute drug-induced hypersensitivity reactions occur quickly following anesthesia induction, mostly in response to neuromuscular blocking agents (NMBA) or antibiotics (3). Despite the presence of an adequate resuscitation setting, the fatality rate of severe perioperative anaphylaxis is estimated at 3-5% (4). Perioperative anaphylaxis diagnosis relies on elevation of circulating mast cell tryptase and positive skin tests to one of the received drugs (5, 6). Additionally, specific IgE against NMBA and antibiotics may be detected but with low sensitivity. The sensitization mechanisms to the drugs are not precisely known, and very few risk factors to develop such a dramatic reaction have been described (7, 8). Accordingly, it is not currently possible to predict nor prevent the development of drug sensitization and occurrence of perioperative anaphylaxis.

A growing body of evidence underlines a link between development of allergic diseases and microbiota (9). Indeed, it has been known for a long time that microbial exposure in a farm environment is highly protective against development of asthma and allergy (10). More recently, studies have linked microbiota alterations with the occurrence of food allergy (11). Lower microbiota richness in early childhood or dysregulation of specific bacteria genera abundance is predictive of the development of food sensitization (12, 13) and allergy to different foods (e.g. eggs or milk) was associated to distinct microbiota alterations compared to nonallergic controls (14, 15). While the mechanisms underlying these associations are not completely understood, murine studies have demonstrated a role for bacterial metabolites in the modulation of the immune system, and in particular to promote or inhibit Th2 immunity and Treg cells (11). These findings have led to clinical trials aiming at developing food tolerance via microbiota manipulation, mostly in milk allergy, with encouraging results (16). However, outside asthma and food allergy, there are very few data on the potential role of microbiota in allergic diseases. In particular, the role of microbiota in perioperative anaphylaxis has never been investigated so far.

In this study, we used 16S rDNA sequencing of plasma specimen from well-characterized individuals with a documented episode of perioperative NMBA-anaphylaxis and matched controls to investigate the potential links between microbiome and clinical and biological features of anaphylaxis.

## Methods

### Patients

The cohort consisted of serum samples collected during the NASA clinical study (NTC01637220). Complete characteristics and results have been described elsewhere (1, 17). From this cohort, we selected 20 patients who experienced fully documented IgE-dependent perioperative NMBA-induced anaphylaxis, and 20 anesthetized controls matched for age (+/- 5 years), sex, infectious status and NMBA received. All patients lived in the same area (Paris region, France). Patients were sampled within 30 min after anaphylaxis, and 2-3 month after (baseline) during an allergology visit. Controls were sampled within 30 min after anesthesia induction. All patients had biological results compatible with mast cell degranulation (using the standard formula [acute mast cell tryptase > 1.2 X basal mast cell tryptase+2µg/L]) and positive skin test to NMBA. The complete clinical and biological characteristics of the patients are described in **Table 1**. The study was approved by the appropriate local ethics authority (committee for the protection of Individuals “Ile-de-France 1”, reference 2012-april-12880).

**Table 1:** Demographical and clinical parameters of the cohort.

	Patients (n=20)	Controls (n=20)	P value
**Age** (mean ± SD)	56.6 ± 12	56.5 ± 18	ns
**Gender, Female**	13 (65%)	12 (60%)	ns
NMBA received			
Suxamethonium	13 (65%)	12 (60%)	ns
Atracurium	6 (30%)	7 (35%)	ns
Rocuronium	2 (10%)	2 (10%)	ns
**Anaphylaxis severity** (severe)	15 (75%)	–	
**Acute Mast cell Tryptase** (µg/L, mean ± SD)	72.4 ± 46	–	
**Basal Mast cell Tryptase** (µg/L, mean ± SD)	4.8 ± 3	–	
**Acute Histamine** (nmol/L, mean ± SD)	94 ± 16	–	
**QAM IgE >0.35kU/L**	8 (40%)	1 (5%)	0.02*

NMBA, Neuromuscular blocking agent; QAM, quaternary ammonium morphine; SD, standard deviation; Ns, non significant.

*Fischer’s exact test.

### Genomic DNA extraction and 16s rDNA quantification

DNA was extracted from the samples using an optimized tissue-specific technique. Total genomic DNA was collected in a final 50μl extraction volume. Total DNA concentrations were determined by UV spectroscopy (Nanodrop^®^, Thermo Scientific).

Real-time PCR amplification was performed using 16S universal primers targeting the V3-V4 region of the bacterial 16S ribosomal gene (Vaiomer universal 16S primers). The qPCR step was performed on a VIIA 7^®^ PCR system (Life Technologies) using Sybr Green technology and the following amplification cycles: hold stage of 10 min at 95°C, then 40 cycles of 15 sec at 95°C, 1 min at 63°C and 1 min at 72°C. The absolute number of copies of 16S rDNA was determined by comparison with a quantitative standard curve of 16S rDNA plasmids generated by serial dilutions of plasmid standards (Vaiomer Universal standard plasmids). The total 16S rDNA present in the samples was measured by qPCR in triplicate and normalized using a plasmid-based standard scale.

### Microbiome sequencing

The microbial population present in the samples has been determined using a previously described metagenomic pipeline (18). Briefly, we used next generation high throughput sequencing of variable regions of the 16S rDNA bacterial gene. The PCR amplification was performed using 16S universal primers targeting the V3-V4 regions of the bacterial 16S ribosomal gene (Vaiomer universal 16S primers). For each sample, a sequencing library was generated by addition of sequencing adapters. The detection of the sequencing fragments was performed with the MiSeq Illumina technology using the 2 x 300 paired-end MiSeq kit V3. The targeted metagenomic sequences from microbiota were analyzed using a previously described bioinformatics pipeline (18). Briefly, after demultiplexing of the bar-coded Illumina paired reads, single read sequences were cleaned and paired for each sample independently into longer fragments. Operational taxonomic units (OTUs) were obtained via single-linkage clustering and taxonomic assignment was performed in order to determine community profiles.

### Statistics

All statistical analyses were performed using the R programming environment, the Prism Software, v9.3 (GraphPad Software Inc.) and the Genesis software 1.8.1 (Gratz University of Technology, Austria). Nonparametric tests, including the Wilcoxon paired rank-sum test, the Mann-Whitney test, and the Kurskal-Wallis test were used for routine statistical analysis where appropriate. Proportions were compared using Fischer’s exact test. Specific statistical analysis of the microbiome data was performed using both custom and freely accessible R packages (18). Only taxa present in >25% of the samples were included in differential abundance analysis. To measure α-diversity, we used the observed richness, the Chao1 index, the Shannon index and the Simpson index. We used Bray-Curtis dissimilarity index as a measure of β-diversity that was subsequently submitted to non-metric multidimensional scaling ordination (MDS) and hierarchical clustering using Ward’s criterion. Detailed comparisons between samples of OTUs relative abundance were performed by linear discriminant analysis effect size [LEfSe, described in (19)]. Correlations between taxa abundances and clinical or biological parameters were evaluated using Spearman’s correlation test and submitted to hierarchical clustering using Ward’s criterion. P-values <0.05 were considered significant.

## Results

### Abundance of 16s rDNA in anaphylaxis patients

**Figure 1:**
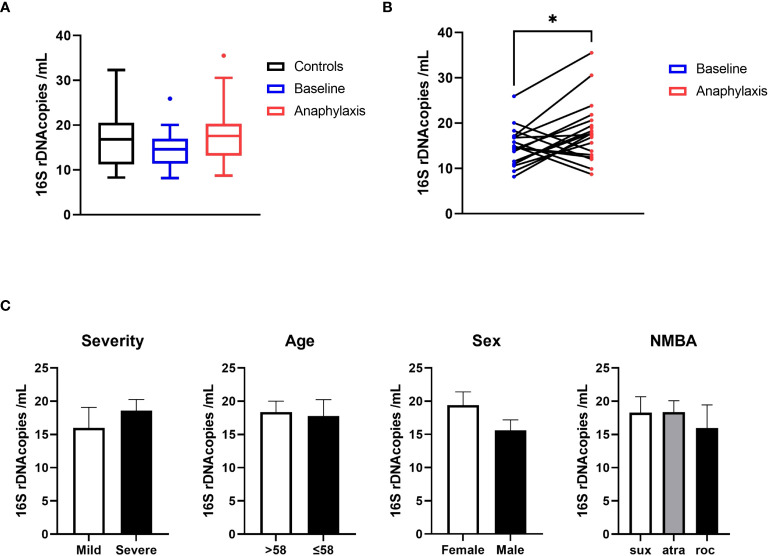
Circulating bacterial 16s RNA is increased during anaphylaxis. **(A)** Quantification of circulating bacterial 16s rDNA by qPCR in anaphylaxis patients during the reaction (red), in the same patients at baseline (blue) and in matched controls (black). **(B)** Pairwise comparison of circulating rDNA in anaphylaxis patients’ samples taken during the reaction (red) and at baseline (blue). **(C)** Comparison of circulating 16s RNA concentrations according to anaphylaxis severity (mild: grade I and II, severe: grade III and IV, Ring and Messmer classification), median age of the cohort, sex, and NMBA received (sux: suxamethonium; atra: atracurium; roc: rocuronium). *p<0.05. Box plot whiskers are 5^th^ and 95^th^ percentile, histograms are mean ± standard error of the mean.

To measure the abundance of circulating bacterial DNA in anaphylaxis patients, we determined the absolute number of circulating 16s rDNA copies in serum samples. Bacterial rDNA was readily detectable for all samples and within quantification range. The rDNA quantity was not globally different between anaphylaxis patients (acute and baseline samples) and controls when the three groups were compared globally using variance analysis (**Figure 1A**). However, when pairwise comparisons were performed for patients between samples collected during anaphylaxis and at baseline, the number of copies was significantly more elevated during anaphylaxis (18.1 vs 14.7 copies/mL, p=0.018; **Figure 1B**). However, this number was not associated to either severity of anaphylaxis, sex, age, or NMBA used (**Figure 1C**). Moreover, there was no significant correlation between the circulating number of 16s rDNA with acute tryptase or histamine concentrations measured during anaphylaxis.

### Diversity of circulating microbiome of anaphylaxis patients

**Figure 2:**
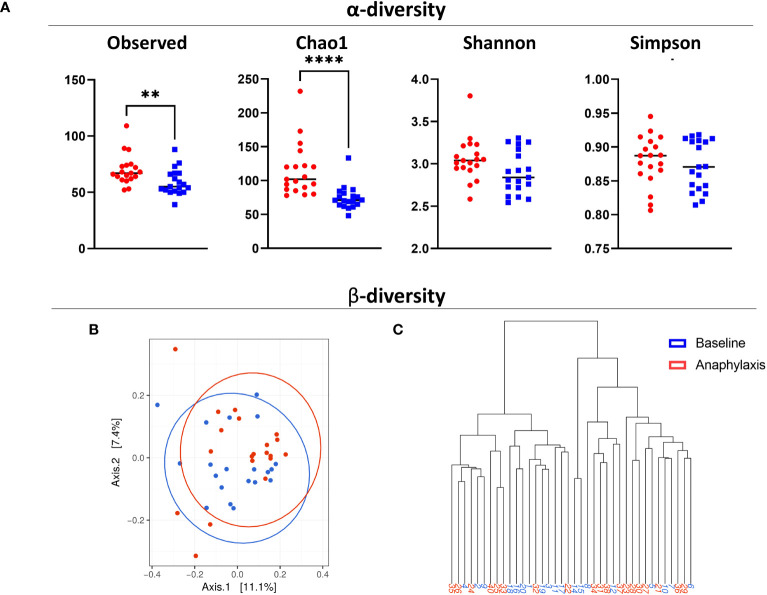
Diversity of circulating microbiota in anaphylaxis patients. **(A)** Measure of α-diversity by direct counts (observed), Chao1 algorithm, Shannon index and Simpson index in anaphylaxis patients at baseline (red) and matched controls (blue). **(B)** Measure of β-diversity by the Bray-Curtis dissimilarity index, representation by multidimensional scaling. **(C)** Dendrogram of hierarchical clustering of samples according to their Bray-Curtis index. ****p<0.0001, **p<0.01.

Next, we compared the microbiome richness and composition between anaphylaxis patients and controls. We first compared alpha and beta diversity in controls and patients samples taken at baseline (**Figure 2A**). Alpha diversity was significantly higher in anaphylaxis patients as shown by the observed number of OTUs and Chao1 index (p=0.0073 and p<0.0001, respectively), which both measure microbiome richness based on different species number. However, there was no difference for Shannon and Simpson indexes which measure both richness and evenness. The beta diversity was calculated by the Bray-Curtis dissimilarity index. Results were subjected to multidimensional scaling ordination (**Figure 2B**) and hierarchical clustering (**Figure 2C**). In both representations, no clear clustering of patients or controls could be observed, meaning that there were no major differences in terms of microbiota composition between the serum of anaphylaxis patients and controls.

Taken together, anaphylaxis patients seem to have a richer circulating microbiota than controls, but no major differences of composition could be detected with global diversity indexes.

### Comparison of OTUs between anaphylaxis patients and controls

**Figure 3:**
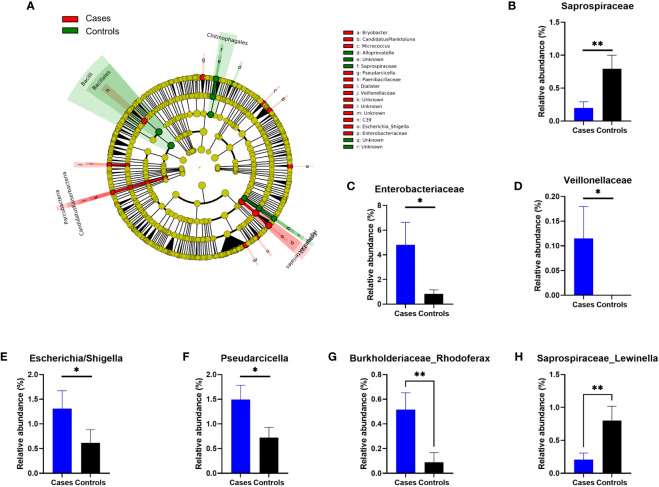
Differences in relative OTUs frequencies between anaphylaxis patients and controls. **(A)** Cladogram representation of OTUs significantly different between anaphylaxis patients (cases) and controls by LEfSe analysis. The five concentric circles are (from inside out): phylae, class, order, family, genus. In red are the OTUs more frequent in anaphylaxis patients, in green the OTUs more frequent in controls. **(B–H)**: Confirmed differences at family and genus levels by non-parametric Mann-Whitney testing. Blue: anaphylaxis patients, black: controls. **p<0.01, *p<0.05. Data are mean ± standard error of the mean.

To analyze in more details the potential differences between both cohorts, we next performed a LEfSe analysis of the frequencies of the OTUs sequenced at phylogenetic levels from genus to phylum (**Figure 3A**). We found several taxa either more frequent in anaphylaxis patients or in controls, and then controlled the differences in relative frequencies by subsequent Mann-Whitney hypothesis tests. We were able to confirm a significant difference for three families (S*aprospiraceae*, p=0,0016; *Enterobacteriaceae*, p=0.018 and *Veillonellaceae*, p=0.04; **Figures 3B–D**) and 2 genera (Enterobacteriaceae/Escherichia-Shigella, p=0.03; Cytophagaceae/Pseudarcicella, p=0.04). A difference was also found for additional genera that were not automatically identified by our algorithm but after manual analysis of sequences could be attributed to *Burkholderiaceae/Rhodoferax*, p=0.006 and *Saprospiraceae/Lewinella*, p=0.0027; **Figures 3E–H**).

### Links between anaphylaxis biological or clinical parameters and OTUs

**Figure 4:**
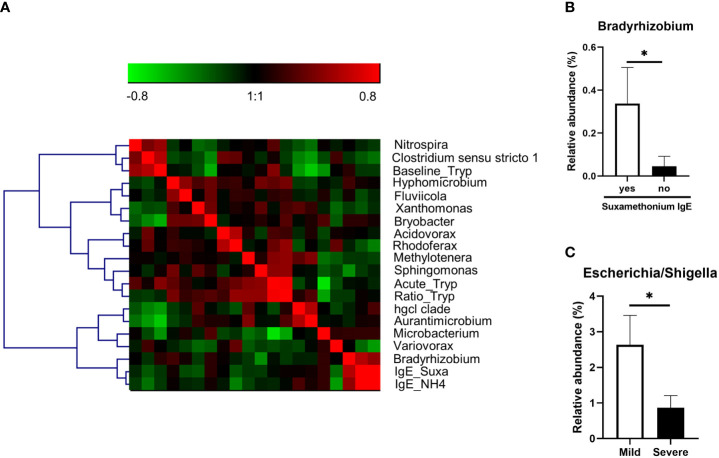
**(A)** Correlation matrix of OTUs (at genus level) significantly correlated to one of the biological parameters (acute tryptase measurement (Acute_Tryp), baseline tryptase measurement (Baseline_Tryp), acute/basal tryptase ratio (Ratio_Tryp), serum anti-suxamethonium IgE (Suxa_IgE), serum anti-NH4 IgE (IgE_NH4). The matrix was submitted to hierarchical clustering using Ward linkage. Red squares are positive correlation and green squares are negative correlations. **(B)** Relative abundance of the genus significantly different between anaphylaxis patients with or without serum anti-suxamethonium IgE **(C)**. Relative abundance of the genus significantly different between mild and severe patients. *: p<0.05. Data are mean ± standard error of the mean.

Finally, we analyzed the association of taxa with biological and clinical parameters of anaphylaxis in patients. For continuous data, we performed a correlation matrix including OTUs at the genus level and tryptase measurements (during anaphylaxis, at baseline, and the ratio of both), and specific IgE against suxamethonium and quaternary ammonium. For better visualization this matrix was submitted to hierarchical clustering (**Figure 4A**). We found several significant correlations with acute tryptase (*Microbacterium, Methylotenera, Rhodoferax, Sphingomonas*), baseline tryptase (*Aurantimicrobium, Bryobacter, Clostridium*, hgcl clade, *Microbacterium, Nitrospira*), and concentration of specific IgE (*Variovorax*, *Bradyrhizobium*) (**Supplementary Table 1**). The latter was confirmed by further analysis using presence of specific IgE to suxamethonium as a binary variable (p=0.01, **Figure 4B**).

Furthermore, when comparing samples from patients with mild versus severe anaphylaxis, we found that *Escherichia/Shigella* genera was significantly more abundant in samples from mild versus severe anaphylaxis patients (p=0.01; **Figure 4C**).

## Discussion

The role of microbiota in the development of allergic disease is a subject of increasing interest. Variations of anaphylaxis frequencies according to geographical origin and diet have been described for a long time (20, 21). More recently, several studies have been able to associate gut or airways microbiota composition with predisposition toward asthma or food allergy. This study is a first attempt at exploring associations between microbiota and drug anaphylaxis.

Using circulating microbiota as a proxy for gut microbiota, we performed 16s rDNA quantitation and sequencing in well-documented IgE-dependent anaphylaxis patients and matched controls. We found significantly more microbial DNA in patients’ samples taken during anaphylaxis as compared to baseline samples, which is usually interpreted as a marker of bacterial translocation from the gut. Bacterial translocation during anaphylaxis has never been described so far. However, a role for histamine in increasing intestinal permeability has been reported (22), making this a possibility. Microbial DNA can be sensed as a signal danger, in particular by neutrophil surface TLR9 (23). Since we demonstrated in a previous study neutrophil activation in anaphylaxis (1), it could be hypothesized that rDNA play a role in sustaining neutrophil activation. Whether this could have a clinical impact in patients remains uncertain since in our study no correlation could be found between circulating rDNA abundance and clinical severity or mast cell tryptase and histamine concentrations.

While we found no major change in microbiota diversity between patients and controls by multivariate analysis, frequencies of several individual OTUs were found to be different between patients and controls. In the literature on associations between microbiota and allergic disease, several taxa have been associated with occurrence of allergic disease (24, 25). For example, food allergy is associated with an increased frequency of *Clostridium* and a decreased frequency of *Bifidobacterium* and *Bacteroidaceae* in the children gut microbiome (13, 26, 27). Additionally, a high frequency of *Clostridium* and *Enterococcus* and a lower frequency of *Faecalibacterium*, *Lachnospira* and *Veillonella* are found very early in the gut of children developing asthma (28). We did not find an association of most of these taxa with NMBA anaphylaxis in our study. However, the literature is mainly focused on risk-prediction of atopy-related diseases in children, which is a very different context from our study on adult-onset drug anaphylaxis, whose link to atopy is debated.

Among the OTUs found to be linked to NMBA anaphylaxis, only *Escherichia/Shigella* and *Veillonellaceae* have previously been associated with allergic diseases. Indeed, *Escherichia/Shigella* frequency is increased in gut of patients with allergic rhinitis (29) and its abundance in airways has been associated with more severe airway obstruction in asthmatic patients (30). *Veillonella* abundance has been linked to several pathologies in humans. For example, it is linked urinary tract infection risk in vagina flora (31), and to caries in oral microbiota (32). Interestingly, its abundance in the airways or in the gut at one month of age is also associated with later asthma development in childhood (33, 34).

Besides allergy, other OTUs that were associated with anaphylaxis in this study have previously been linked to diseases. *Pseudarcicella* was depleted in plasma of patients with cirrhosis as compared to healthy controls (35), and also increased in lung of dogs with idiopathic pulmonary fibrosis (36). The relative frequency of its family, *Cytophagaceae*, was found to be increased in plasma of patients with Takayasus’ arteritis as compared to healthy controls (37). Finally, *Lewinella* frequency in blood microbiome has been associated with clinical response to nivolumab in NSCLC (38). *Rhodoferax* and *Bradyrhizobium* have never been linked to a disease, in fact only *Bradyrhizobium* has already been reported in humans, as a possible component of the ocular surface microbiota (39). These bacteria being abundantly present in the environment, their presence in human microbiome and association with diseases remain to be verified.

This study presents some limitations. First, circulating microbiome analysis is a relatively new method with technical limitations, in particular the very low amount of circulating microbial rDNA available, and have been as such criticized for lack of specificity (40). The technique we used from the Vaiomer company has been specifically designed to address these criticism and limit the risk of contamination (18, 41, 42). However, our findings should be verified on stool samples that are much richer and thus easier to analyze. Second, the number of samples analyzed is low compared to other microbiota studies. NMBA anaphylaxis is a rare disease, and we chose to favor a homogenous population with fully characterized IgE-dependant anaphylaxis (i.e. with elevated circulating tryptase and histamine during anaphylaxis and positive skin test to the NMBA received) thus limiting the number of eligible patient in our cohort. While this limits the significance of our results, we believe our work represents a first step in exploring microbiota of patients with NMBA anaphylaxis.

In conclusion, we did not find a major difference in terms of microbiota composition between anaphylaxis patient and controls. However, circulating rDNA was more abundant during anaphylaxis than at baseline, and some OTUs were associated with anaphylaxis patients, prompting further studies to confirm these findings in different settings. Understanding the precise influence of the disease-associated bacteria on the immune system will be necessary to broaden our understanding of drug anaphylaxis pathophysiology and define predisposition markers.

## Data availability statement

The raw sequencing data from this study is available on The Sequence Read Archive (SRA) under the NCBI BioProject ID PRJNA1061678.

## Ethics statement

The studies involving humans were approved by French committee for the protection of Individuals “Ile-de-France 1”. The studies were conducted in accordance with the local legislation and institutional requirements. The participants provided their written informed consent to participate in this study.

## Author contributions

LdC, SC-M, A-MC, and PM designed the study. LdC, DC, AG-C, and VG performed data collection and experiments. LdC, DC, DL, PM, A-MC, and SC-M analyzed the results. LdC drafted the manuscript. All authors contributed to the article and approved the submitted version.
