# Supplementary material for: Circulating microbiome analysis in patients with perioperative anaphylaxis
Source: Front Immunol. 2024 Jan 11;14:1241851. doi: 10.3389/fimmu.2023.1241851 (PMC10808669; doi:10.3389/fimmu.2023.1241851)
Supplement: Supplementary file 1 [file Table_1.docx]

|  | **Acute_Tryp** | **Baseline_Tryp** | **Ratio_Tryp** | **IgE_Suxa** | **IgE_NH4** |
| --- | --- | --- | --- | --- | --- |
| **Aurantimicrobium** | -0.0592 | **-0.6325** | 0.2501 | 0.0624 | 0.0756 |
| **Bradyrhizobium** | -0.0546 | 0.128 | -0.1729 | **0.563** | **0.4566** |
| **Bryobacter** | 0.0236 | **-0.5489** | 0.2635 | 0.1356 | 0.0672 |
| **Clostridium** | 0.0609 | **0.5674** | -0.1942 | -0.2797 | -0.3579 |
| **hgcI clade** | -0.2221 | **-0.4796** | 0.1157 | 0.0315 | 0.2279 |
| **Methylotenera** | 0.4162 | -0.0629 | **0.4495** | -0.1684 | -0.2077 |
| **Microbacterium** | **-0.7163** | **-0.4635** | **-0.5169** | 0.1574 | 0.1613 |
| **Nitrospira** | 0.2687 | **0.4707** | -0.164 | -0.1172 | -0.1807 |
| **Rhodoferax** | 0.3277 | 0.0221 | **0.4587** | -0.2623 | -0.4025 |
| **Sphingomonas** | 0.4373 | 0.0505 | **0.4528** | -0.1847 | -0.1011 |
| **Variovorax** | -0.247 | 0.0108 | -0.1818 | -0.3337 | **-0.49** |
|  |  |  |  |  |  |
|  |  |  |  |  |  |
|  | p<0.05 | p<0.01 | p<0.001 |  |  |
|  |  |  |  |  |  |

**Supplementary Table 1**

| **Correlation of taxa abundance with biological parameters**  Acute_Tryp: acute tryptase; Baseline_Tryp: baseline tryptase; Ratio_Tryp: ratio between acute and basal tryptase; IgE_Suxa: specific IgE to suxamethonium; specific IgE to quaternary ammonium**.** |
| --- |
